# Bisphenol A and 17β-Estradiol Promote Arrhythmia in the Female Heart via Alteration of Calcium Handling

**DOI:** 10.1371/journal.pone.0025455

**Published:** 2011-09-27

**Authors:** Sujuan Yan, Yamei Chen, Min Dong, Weizhong Song, Scott M. Belcher, Hong-Sheng Wang

**Affiliations:** Department of Pharmacology and Cell Biophysics, University of Cincinnati College of Medicine, Cincinnati, Ohio, United States of America; Brigham & Women's Hospital - Harvard Medical School, United States of America

## Abstract

**Background:**

There is wide-spread human exposure to bisphenol A (BPA), a ubiquitous estrogenic endocrine disruptor that has been implicated as having potentially harmful effects on human heart health. Higher urine BPA concentrations have been shown to be associated with cardiovascular diseases in humans. However, neither the nature nor the mechanism(s) of BPA action on the heart are understood.

**Methodology/Principal Findings:**

The rapid (<7 min) effects of BPA and 17β-estradiol (E2) in the heart and ventricular myocytes from rodents were investigated in the present study. In isolated ventricular myocytes from young adult females, but not males, physiological concentrations of BPA or E2 (10^−9^ M) rapidly induced arrhythmogenic triggered activities. The effects of BPA were particularly pronounced when combined with estradiol. Under conditions of catecholamine stimulation, E2 and BPA promoted ventricular arrhythmias in female, but not male, hearts. The cellular mechanism of the female-specific pro-arrhythmic effects of BPA and E2 were investigated. Exposure to E2 and/or BPA rapidly altered myocyte Ca^2+^ handling; in particular, estrogens markedly increased sarcoplasmic reticulum (SR) Ca^2+^ leak, and increased SR Ca^2+^ load. Ryanodine (10^−7^ M) inhibition of SR Ca^2+^ leak suppressed estrogen-induced triggered activities. The rapid response of female myocytes to estrogens was abolished in an estrogen receptor (ER) β knockout mouse model.

**Conclusions/Significance:**

Physiologically-relevant concentrations of BPA and E2 promote arrhythmias in a female-specific manner in rat hearts; the pro-arrhythmic actions of estrogens are mediated by ERβ-signaling through alterations of myocyte Ca^2+^ handling, particularly increases in SR Ca^2+^ leak. Our study provides the first experimental evidence suggesting that exposure to estrogenic endocrine disrupting chemicals and the unique sensitivity of female hearts to estrogens may play a role in arrhythmogenesis in the female heart.

## Introduction

Estrogenic endocrine disrupting chemicals (EDCs) are a structurally diverse group of compounds that mimic, or antagonize the effects of endogenous estrogens. A particularly significant example of estrogenic EDCs to human health is the nearly ubiquitous xenoestrogen bisphenol A (BPA). BPA is structurally similar to the potent non-steroidal synthetic estrogen diethylstilbestrol. As one of the highest produced synthetic chemicals worldwide, BPA is used extensively in the production of polycarbonate plastic and epoxy resins that are found in a wide range of consumer products such as food containers, food cans, water bottles, baby bottles, dental sealants and water pipes. Bioactive BPA is released from epoxy resins and polycarbonate food and beverage containers, especially after exposure to elevated temperatures [1]. Consequently, there is broad human exposure to BPA [2]. It has been shown that BPA is present at detectable levels in urine, which are considered the appropriate body fluid for assessing BPA exposure, of over 90% of individuals examined in the US population [2], [3], [4], [5].

There is significant public, scientific and regulatory interest in elucidating the impact of BPA exposure on human health. Experimental evidence has demonstrated potential links between BPA exposure and cancer, obesity, diabetes, and disorders of the reproductive, neuroendocrine and immune systems in a variety of cellular and animal models [6]. The dose range in the animal studies demonstrating BPA-induced pathogenesis correlates with the BPA concentrations for human exposure [2], [7], [8], [9]. However, despite the increasing recognition that the endocrine disrupting activities of BPA may have adverse health impact, the effect(s) of environmentally-relevant concentrations of BPA remains controversial, and the impact of BPA in the heart is unknown. Retrospective epidemiologic investigation of health effects associated with BPA exposure suggests that higher urine BPA concentrations are associated with cardiovascular disease and other diseases in the US population [10], highlighting the potentially important influence of BPA exposure on cardiac pathophysiology.

Cardiovascular disease is a leading cause of mortality in developed countries, for both men and women. Estrogen has strong influences on the sexually-dimorphic baseline physiology of the heart, and on myocardial responses associated with various cardiac pathophysiological conditions including hypertrophy, failure, ischemic injury, and arrhythmias [11], [12], [13], [14]. The regulatory mechanisms of estrogen's actions on the myocardium are complex. In addition to regulation of gene expression through nuclear hormone receptor activity, estradiol and BPA can elicit cell-specific responses through activation of intracellular signaling pathways associated with membrane-localized estrogen receptors [12], [13], [15]. These effects occur rapidly within seconds to minutes and are independent from the “classical” nuclear hormone receptor gene regulatory pathways. In cardiac myocytes, limited evidence shows that 17β-estradiol (E2), often at supra-physiological concentrations, rapidly affects membrane ionic conductance, Ca^2+^ handling, and excitation-contraction coupling [12], [15], [16], [17], [18]. The effects of BPA on the myocardium, including its rapid action, are entirely unknown.

The aims of the present study were to investigate the rapid, sex-specific effects of physiologically-relevant concentrations of BPA and E2 on rodent cardiac myocytes and hearts, with a focus on the arrhythmogenic effect of estrogens, and to determine the underlying cellular mechanism of the rapid actions of estrogens.

## Results

### BPA and E2 Rapidly Promote Triggered Activities in Female Rat Myocytes

**Figure 1:**
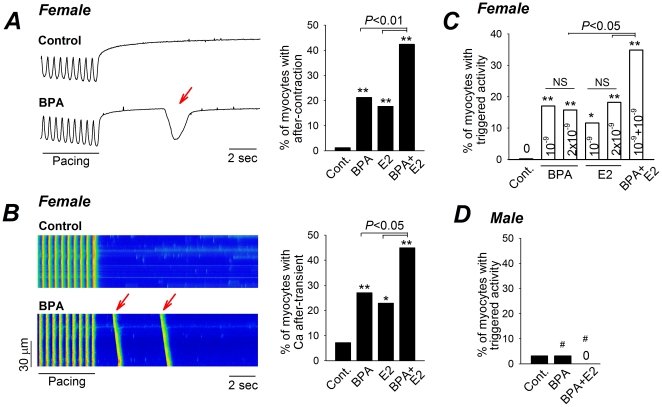
Acute exposures to BPA and E2 promote triggered activities in female rat ventricular myocytes. (**A**) Left, representative contraction traces of female rat myocytes elicited by pacing under control and upon acute exposure to 10^−9^ M BPA. Arrow indicates spontaneous after-contraction (i.e., triggered activity) following pacing. Right, percentages of myocytes with after-contractions under various conditions. N = 52–87 myocytes. (**B**) Left, representative Ca^2+^ transients in female rat myocytes elicited by pacing under control and upon acute exposure to 10^−9^ M BPA. Arrows indicate spontaneous Ca^2+^ after-transients (i.e., triggered activity) following pacing. Right, percentages of myocytes with after-transients under various conditions. N = 35–74 myocytes. (**C**) Percentages of female myocytes with triggered activities under BPA or E2 alone, at 10^−9^ and 2×10^−9^ M, and under a mixture of BPA and E2, both at 10^−9^ M; n = 42–66. (**D**) Percentages of male ventricular myocytes with triggered activities under control, 10^−9^ M BPA and a mixture of BPA and E2, both at 10^−9^ M, N =  32 myocytes for all groups. #: P>0.1; *: P<0.05; **: P<0.01 vs. control in a χ^2^ test. NS (not significant): P>0.1.

Triggered activities are abnormal excitations of cardiac myocytes “triggered” by preceding impulses, and are a key arrhythmogenic mechanism in the heart [19], [20]. The effect of BPA or E2 exposure on triggered activities, measured as spontaneous after-contractions following repeated pacing, was assessed in isolated ventricular myocytes from each sex (Figure 1A). In myocytes from female rat hearts, brief exposure (2–7 min) to BPA or E2 (10^−9^ M) resulted in detectable after-contractions in 21% and 18% of the myocytes, respectively. By comparison, only 1% of myocytes displayed after-contraction under control conditions. Exposure to a mixture of 10^−9^ M BPA and 10^−9^ M E2 significantly increased the percentage of cells with after-contractions to 42%. Measurements of triggered activity as spontaneous Ca^2+^ transients following pacing yielded similar results (Figure 1B). BPA or E2 alone (10^−9^ M) induced spontaneous Ca^2+^ transients in 27% and 23% of female rat myocytes, respectively, and in 45% of myocytes when added as 10^−9^ M equal molar mixture.

In contrast to the robust effects of the BPA and E2 mixture, increasing E2 or BPA concentration two-fold from 1×10^−9^ M to 2×10^−9^ M did not increase the induced responses in female rat myocytes (Figure 1C). This finding suggests that the amplified effects observed in response to an equal molar mixture of BPA and E2 were not the simple result of an additive increase in estrogen concentration. The pro-arrhythmic cardiac sensitivity to BPA and/or E2 was female-specific; exposure of ventricular myocytes from male rats to BPA alone or BPA combined with E2 did not result in any increase in triggered activities (Figure 1D).

### BPA and E2 Promote Ventricular Arrhythmias in Female Rat Hearts

**Figure 2:**
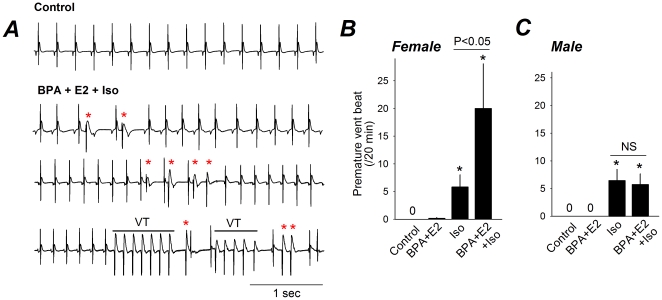
Acute exposures to BPA and E2 promote ventricular arrhythmias in female rat hearts. (**A**) Representative surface ECG traces from female hearts under control or in the presence of BPA and E2, both at 10^−9^ M, and 10^−8^ M isoproterenol. Asterisks indicate premature ventricular beats (PVBs). VT: ventricular tachycardia. (**B**) Average frequency of PVBs in female hearts under various conditions. N = 6–11 hearts. (**C**) Average frequency of PVBs in male hearts under various conditions. N = 7–9 hearts. Error bars are SEM. *: P<0.05 vs. control in a t-test. NS (not significant): P>0.1.

The effect of BPA and E2 on arrhythmias in rat hearts was examined by surface electrocardiogram (Figure 2). In both female and male rat hearts, arrhythmias were absent under control conditions and very infrequent following exposure to 10^−9^ BPA and E2. In female rat hearts under catecholamine-induced stress [isoproterenol (Iso), 10^−8^ M], exposure to BPA and E2 markedly increased the frequency of ectopic ventricular beats from 5.8±2.2 to 20±8 per 20 min (*P*<0.05; Figure 2A and 2B). In addition, BPA and E2 exposure in the presence of Iso resulted in episodes of non-sustained ventricular tachycardia (VT) in one of the six female hearts analyzed (Figure 2A). The arrhythmogenic effects of BPA and E2 were female-specific; in male rat hearts, exposure to E2 and BPA did not increase the frequency of premature ventricular beats compared with Iso alone (Figure 2C), or induce other forms of identifiable arrhythmia.

### BPA and E2 Alter Myocyte Calcium Handling

**Figure 3:**
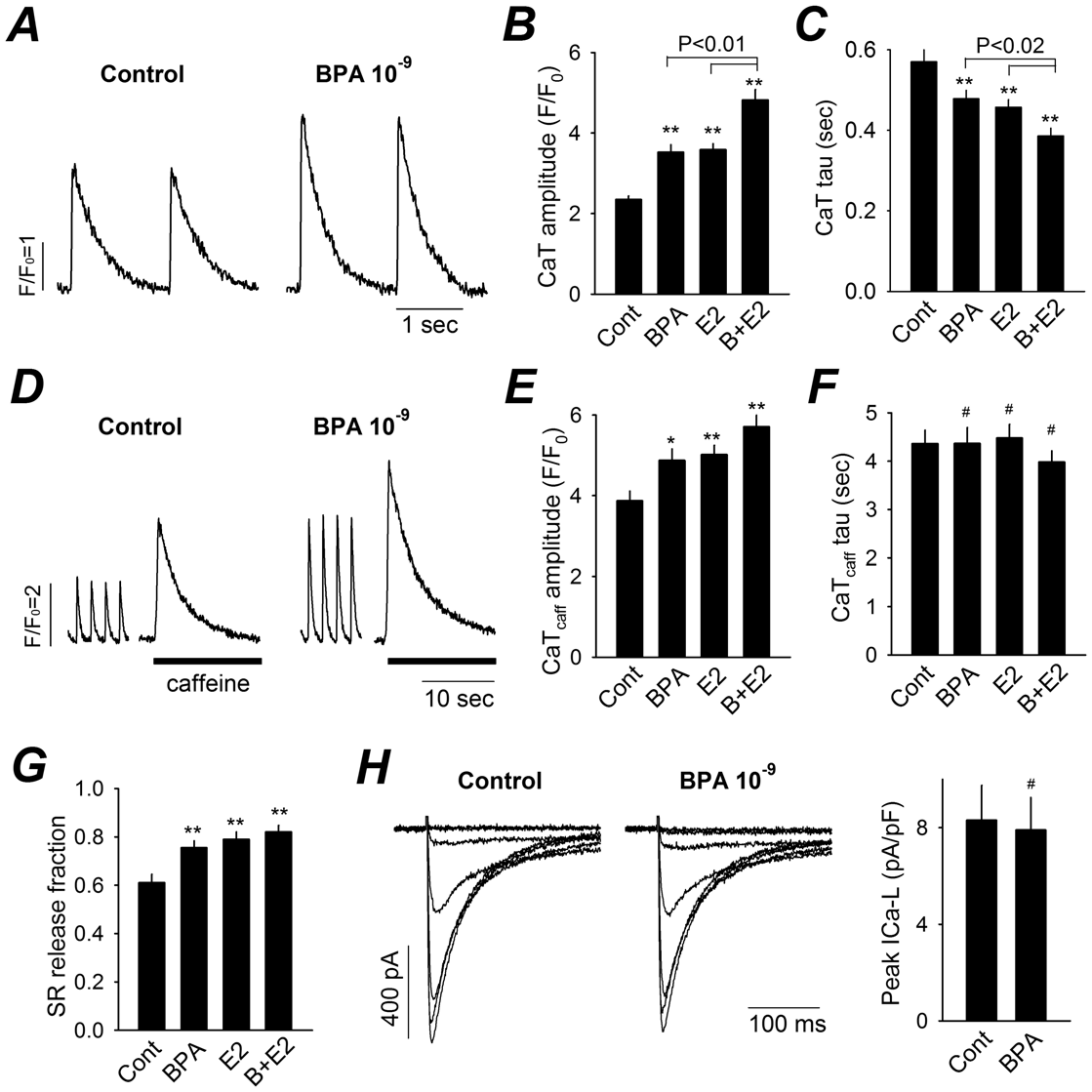
Effect of BPA and E2 on Ca^2**+**^ handling in female rat myocytes. (**A**) Representative Ca^2+^ transients (CaT) elicited by steady-state field stimulation under control and in the presence of BPA. (**B**) and (**C**) Average data on CaT amplitude and decay time constant under control (Cont), 10^−9^ M BPA or E2, and 10^−9^ M BPA plus 10^−9^ M E2 (B+E2). N = 27–42. (**D**) Representative Ca^2+^ transients induced by 10 mM caffeine following repeated field stimulation. Black bars indicate exposure to caffeine. (**E**) and (**F**) Average data on caffeine-induced CaT (CaT_caff_) amplitude and decay time constant (n = 14–19). Treatment labels are the same as (B) and (C). (**G**) SR Ca^2+^ release fractions under various conditions. Treatment labels are the same as (B) and (C). N = 14–19. (**H**) Left, representative L-type Ca^2+^ currents recorded under control and upon exposure to BPA. Shown are traces elicited by voltage steps from -40 to +20 mV in 10 mV increment, from a holding potential of -50 mV. Right, average peak I_CaL_ densities before and after 2–7 min exposure to 10^−9^ M BPA. N = 7. Error bars are SEM. #: P>0.1; *: P<0.05; **: P<0.01 vs. control in a one-way ANOVA or t-test.

Abnormal Ca^2+^ handling plays an important role in the development of triggered activities in the heart [20], [21]. The effects of BPA and E2 on Ca^2+^ handling in female rat myocytes were examined. BPA and E2 alone, and more so when combined, significantly increased the amplitude of field-stimulated Ca^2+^ transients (Figure 3A and 3B). The average peak Ca^2+^ transient amplitude (F/F_0_ ratio) was 2.3 in control myocytes, and 3.5 and 3.6 in myocytes exposed to BPA or E2, respectively. An additional increase in peak Ca^2+^ transient amplitude to 4.8 was observed in myocytes exposed to BPA and E2. The decay rate of the Ca^2+^ transients was also increased (Figure 3C), indicating enhanced SR Ca^2+^ reuptake [20]. Sarcoplasmic reticulum (SR) Ca^2+^ load was assessed as the amplitude of Ca^2+^ transients induced by caffeine (an agonist of ryanodine receptors, or RyRs) (Figure 3D). Consistent with enhanced SR Ca^2+^ reuptake, BPA and E2 markedly increased SR Ca^2+^ load on a beat-to-beat basis (Figure 3D and 3E). The decay rate of the caffeine-induced Ca^2+^ transient, an indication of Ca^2+^ extrusion from the cytosol, was not affected (Figure 3F). The fraction of SR Ca^2+^ release on a beat-to-beat basis, calculated from the ratio of field-stimulated and caffeine-induced Ca^2+^ transients, was also increased following BPA and E2 exposures (Figure 3G). However, the L-type Ca^2+^ current was not affected by BPA (Figure 3H). As a whole, these results indicate that a key effect of estrogen exposure on myocyte Ca^2+^ handling is enhanced SR Ca^2+^ release and reuptake.

### BPA and E2 Promote Triggered Activities by Increasing SR Calcium Leak

**Figure 4:**
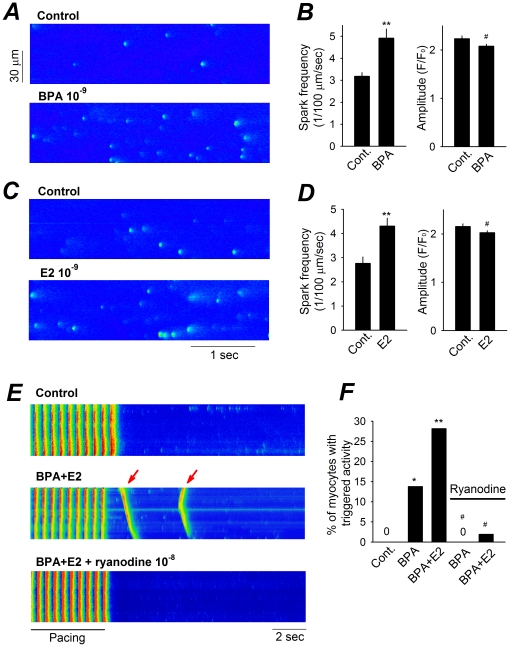
Effect of BPA and E2 on SR Ca^2**+**^ leak in female rat myocyte. (**A**) Ca^2+^ sparks recorded from a myocyte under control and upon rapid exposure to BPA. (**B**) Average data on Ca^2+^ spark frequency (left) and amplitude (right) under control and after exposure to 10^−9^ M BPA. Data are averages of 137 sparks (Control) and 214 sparks (BPA) from 16 myocytes. (**C**) Ca^2+^ sparks recorded from a myocyte under control and upon rapid exposure to E2. Average effects of 10^−9^ M E2 on spark properties are shown in (**D**). Data are averages of 162 sparks (Control) and 230 sparks (E2) from 16 myocytes. (**E**) Representative Ca^2+^ transients elicited by pacing under control, 10^−9^ M BPA plus 10^−9^ M E2, and BPA plus E2 in the presence of ryanodine. Arrows indicate spontaneous Ca^2+^ after-transients following pacing. (**F**) Percentages of myocytes with triggered activities under various conditions. N = 23–52 myocytes. #: P>0.1; *: P<0.05; **: P<0.01 vs. control in a t-test or one-way ANOVA.

The effect of BPA and E2 on SR Ca^2+^ release was further assessed by analysis of Ca^2+^ sparks. Ca^2+^ sparks, which represent local and quantal release of SR Ca^2+^ through clusters of RyRs [22], were examined in quiescent myocytes (Figure 4). Acute exposure to 10^−9^ M BPA significantly increased the average Ca^2+^ spark frequency in female rat myocytes from 3.2 to 4.9 sparks/100 µm/s (Figure 4A and 4B), with no change in peak amplitude (Figure 4B) or spatial/temporal properties (data not shown). Likewise, E2 rapidly increased Ca^2+^ spark frequency without altering spark amplitude (Figure 4C and 4D). Spark frequency is affected by both RyR open probability and SR Ca^2+^ load. Neither E2 nor BPA affected SR load in quiescent myocytes in the absence of active Ca^2+^ cycling (data not shown). Therefore, the observed increase in Ca^2+^ release is most likely the result of increased RyR opening. The role of increased spontaneous SR Ca^2+^ release, or “Ca^2+^ leak”, in the development of estrogen-induced triggered activities was examined using ryanodine [23]. Blockade of RyRs with 10^−7^ M ryanodine did not affect field-stimulation elicited Ca^2+^ transients (Figure 4E), but suppressed spontaneous excitations induced by either BPA alone or BPA combined with E2 (Figure 4E and 4F).

### Ablation of ERβ Abolished Rapid Effects of BPA and E2

**Figure 5:**
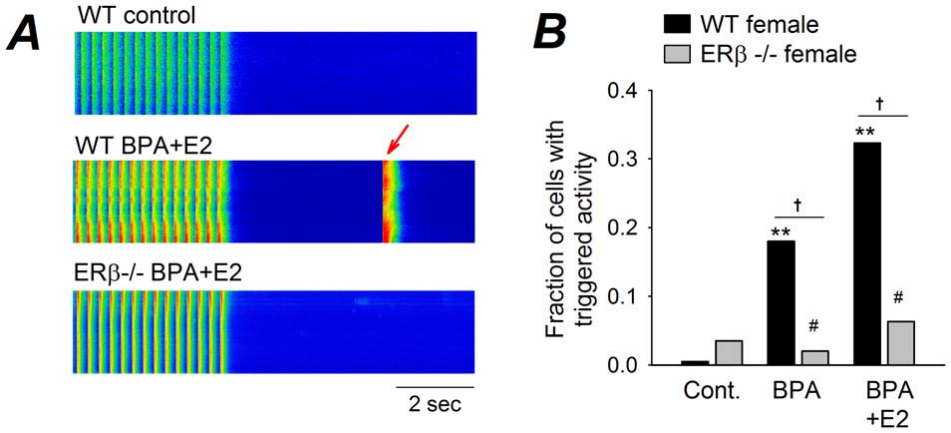
Rapid effects of estrogens in female mouse myocytes are mediated by ERβ signaling. (**A**) Representative recordings of Ca transients elicited by repeated pacing from WT female mouse myocytes under control and in the presence of BPA plus E2 (both 10^−9^ M), and from female ERβ knockout (KO) myocytes in the presence of BPA plus E2. Arrow indicates spontaneous Ca transient. (**B**) Percentages of myocytes with triggered activities under control, 10^−9^ M BPA, and BPA plus E2, both 10^−9^ M. N = 57–86. #: P>0.1; **: P<0.01 vs. control in a χ^2^ test; †: P<0.01. P>0.1 for WT vs KO under control condition, and among the three groups (control, BPA and BPA + E2) for KO cells.

Estradiol exerts its biological effects by activation of the nuclear hormone receptors ERα and ERβ. The role of ERβ in mediating the sensitivity of female rodent myocytes to estrogens was examined using an ERβ knockout mouse model [24]. Similar to the effects observed in female rat myocytes, BPA and E2 rapidly promoted the development of triggered activity in female wild type mouse myocytes; by contrast, the stimulatory effects of estrogens on triggered activities were completely abolished in ERβ^-/-^ female mouse myocytes (Figure 5A and 5B).

## Discussion

Endogenous estrogens are well recognized to play important roles in the regulation and maintenance of sex-specific differences in cardiac physiology and pathophysiology; however, little is known about the cardiac effects of environmental estrogenic EDCs, such as the ubiquitous BPA, and their interactions with endogenous estrogens. In the present study, we show that physiologically-relevant concentrations of BPA and E2 have female-specific pro-arrhythmic effects in rodent cardiac myocytes and whole hearts. The effects of BPA and E2 are mediated by ERβ-signaling, through rapid alteration of myocyte Ca^2+^ handling, particularly by increase in Ca^2+^ leak from the SR. Our animal study results, for the first time, suggest a potential contributing role of BPA (and potentially other estrogenic EDCs) in the development of cardiac arrhythmias in the female heart.

When assessed using representative urine samples, BPA was detected in over 90% of the US population with mean concentration in the low ng/mL, or low-nanomolar range [3], [4]. As an indication of internal exposure, unconjugated BPA in plasma, serum or blood was detected in most individuals in various sampled populations, with mean concentration also in the low-nanomolar range [2]. Initial dose-response analysis of the rapid effects of BPA and E2 in female myocytes was performed using myocyte contractility as an index. For each compound, the dose-response curve had an inverted-U shape; a stimulatory effect on contractility was observed at doses as low as 10^−12^ M, with 10^−9^ M being the most efficacious (data not shown). Therefore, based on experimental considerations, estimated human exposure levels, and BPA's pharmacodynamic profile, 10^−9^ M was selected for further analysis. We demonstrated here that in ventricular myocytes from female but not male rats, exposure to 1 nM BPA or E2, and particularly BPA combined with E2, markedly increased the percentage of myocytes with spontaneous excitations following repeated pacing. These triggered activities are indicative of delayed after-depolarizations, or DADs, a myocyte phenomenon that is recognized as a key non-reentrant arrhythmogenic mechanism [19], [20], [21]. Delayed after-depolarizations are generated under SR Ca^2+^ overload condition and are the result of diastolic spontaneous Ca^2+^ release from the SR. Such spontaneous SR Ca^2+^ release triggers Ca^2+^ extrusion by the Na^+^/Ca^2+^ exchanger (NCX); the activity of the electrogenic NCX generates a depolarizing current, leading to excitation of the myocyte during the diastole. These ectopic excitations can propagate through the myocardium and initiate arrhythmia events [21]. Key factors in the development of DADs include increased SR Ca^2+^ load, and abnormal SR Ca^2+^ release (i.e., SR Ca^2+^ leak). In particular, aberrant RyR opening and diastolic SR leak have been shown to be a central factor in the development of DADs and lethal ventricular arrhythmias under disease conditions such as heart failure [25], [26]. Consistent with the mechanism of DAD development, we found that in female rat myocytes, rapid exposure to BPA or E2 markedly increased SR Ca^2+^ reuptake, SR load, and the fraction of SR Ca^2+^ release on a beat-to-beat basis. Further, E2 and BPA significantly increased the frequency of spontaneous Ca^2+^ sparks, likely as a result of increasing RyR open probability. Supporting a key role of abnormal SR leak in the pro-arrhythmic effect of BPA in female myocytes, we showed that suppression of RyR release by ryanodine, while not affecting normal Ca^2+^ transients, abolished DADs triggered by BPA alone or BPA combined with E2.

Unlike the robust response of female myocytes to BPA and E2, neither compound induced arrhythmias under baseline conditions. However, with β-adrenergic receptor stimulation, BPA combined with E2 resulted in a marked increase in ventricular arrhythmic events, including premature ventricular contractions and ventricular tachycardia in female rat hearts. The discrepancy between the effects of estrogens at the myocyte and whole heart levels likely reflects the fact that only a fraction of the single myocyte-level abnormal electrical activities may propagate and result in arrhythmic events. How myocyte-level triggered activities lead to whole-myocardium arrhythmias, particularly sustained arrhythmias, is complex and not fully understood. Catecholamines affect myocyte mechanics by enhancing Ca^2+^ influx through the L-type Ca^2+^ channels, SR reuptake (and consequently SR Ca^2+^ load), and RyR activities [20], [27]. These effects favor SR overload and abnormal SR Ca^2+^ release, and likely potentiate the pro-arrhythmic actions of these estrogens. Our results suggest that BPA exposure may become a particularly significant factor for arrhythmogenesis in females under stress conditions, and possibly target females with existing cardiac pathophysiological conditions that provide the substrate for arrhythmogenesis.

The rapid effects of BPA on DAD development and Ca^2+^ handling appeared to be amplified in the presence of E2. This intriguing experimental effect raises the possibility that the pro-arrhythmic effect of BPA may be more pronounced in females with higher levels of endogenous estrogens, such as during pregnancy. Bisphenol A has been shown to have additive or synergistic effects with E2 or xenoestrogens in various cell types or systems in other studies [28], [29], [30], [31]. These results contrast with the findings in rat cerebellum, where BPA alone mimics the rapid effects of E2, but acts as an antagonist when combined with E2 [32]. The mechanism(s) underlying the interaction between BPA and E2 is currently unknown. The amplified effect of BPA and E2 in myocytes cannot be mimicked by doubling the dose of BPA or E2 (Figure 1C), suggesting that BPA elicits rapid effects via a mechanism that more complex than can be explained by considering E2 and BPA as equivalent rapid signaling estrogens. Analyses of the actions of BPA and E2 in female myocytes indicate that these effects are mediated via differential intracellular signaling through ERα and ERβ receptor (unpublished results). The known difference in affinity and binding properties of BPA and E2, and the differences in their signaling effects could account for the complex actions of E2 and BPA response. The interaction of BPA and E2-mediated effects observed here support further the notion that estrogenic actions are diverse and tissue and sex-specific; the endocrine disruption actions of estrogenic EDC should be assessed in the context of endogenous estrogens and possibly higher order mixtures of other estrogenic EDCs.

Bisphenol A elicits its biological responses through activation of ER or ER-like receptors. Both ERα and ERβ, including those localized to the membrane, have been shown to be expressed in cardiomyocytes from species including rat and human [13], [33], [34]. Our results using the ERβ-/- mouse model strongly suggest that ERβ-mediated signaling plays a central role in the rapid actions of BPA and estrogen in female rodent cardiac myocytes. Consistent with this notion, we found that in female rat myocytes, the ERβ agonist DPN mimicked the rapid effects of BPA and E2, and the ERβ selective antagonist PHTPP (but not the ERα antagonist MPP) abolished the rapid effects of estrogens; G1, an agonist of the orphan G-protein coupled receptor GPR30, had no detectable effect in female myocytes (data not shown). A similar key role of ERβ in mediating the rapid effects of estrogenic EDCs has been described in cerebellar granular cells [35]. Membrane ERs have been shown to activate kinases including protein kinase A and C [36], [37], which are known to modulate various elements involved in myocyte Ca^2+^ handling [20], [27] and may play important roles in mediating the rapid action of BPA and E2 in female rodent myocytes.

Despite the perception that female hormones protect women from cardiovascular disease, disease of the cardiovascular system is the leading cause of mortality for women in the US. According to the American Heart Associate statistics, since 1984 more women than men have died of cardiovascular disease every year in the US; 53% of total cardiovascular disease deaths occur in women. There is well-recognized sexually dimorphism in cardiovascular disease, including cardiac arrhythmias. Compared with men, women have a lower incidence of sudden cardiac death owning to the protective effect of estrogens on coronary artery disease, and have lower risk of atrial fibrillation; however, women have higher rates of long QT syndrome, sudden cardiac death in the absence of coronary artery disease, and ventricular arrhythmias post myocardial infarction [14], [15], [38], [39], [40]. Incidence of arrhythmias increases during pregnancy, and in women taking oral contraceptives [14], [38], suggesting that female sex hormones may contribute to arrhythmogenesis. Our study provides the first experimental evidence suggesting that exposure to estrogenic EDCs like BPA, and the unique sensitivity of female hearts to estrogens, may play a role in arrhythmogenesis in the female heart. Elucidation of the cardiac effects of endogenous estrogen, environmental estrogenic EDCs, and their interactions is important for assessing the unique cardiovascular benefits and/or risks of both sexes, and may facilitate the development of therapeutic measures that protect against the cardiac risks associated with estrogenic EDC exposure.

## Materials and Methods

### Reagents

Reagents and solvents used were of the highest purity available. All aqueous solutions were prepared using BPA-free water (18 MΩ; <6 ppb total oxidizable organics; Millipore A10 system). Dimethyl sulfoxide (Chromasolv Plus, HPLC ≤99.7%; batch no. 00451HE), bisphenol A (BPA), CAS 80-05-7, >99%, 23,965-8; lot Cl03105ES, isoproterenol hydrochloride, CAS 5984-95-2 and BSA, CAS 9048-46-8, >98% (batch no. 078K0730) were from Sigma-Aldrich (St. Louis, MO). 1,3,5 (10)-estratriene-3,17β-diol (17β-estradiol, 17β-E2; E0959, batch B0356) was from Steraloids (Newport RI). Other chemicals were from Sigma-Aldrich unless otherwise stated.

### Animals

All animal procedures were done in accordance with protocols approved by the University of Cincinnati Institutional Animal Care and Use Committee and followed recommendations of the Panel on Euthanasia of the American Veterinary Medical Association.

Adult Sprague-Dawley rats (200–250 grams; Harlan; Indianapolis, IN) and ERβ (Esr2) knockout mice (ERβ^-/-^; Jackson Laboratory; Bar Harbor, ME) were utilized as non-surviving sources of ventricular tissue and hearts. Animals were anesthetized with sodium pentobarbital (80 mg/kg, i.p.) and heart rapidly dissected for myocyte dissociation. The ERβ^-/-^ mice and age matched wild type littermates were reared at the University of Cincinnati laboratory animal facility and used at 6–8 weeks of age. This ensured that the hearts were free of abnormality associated with systemic effects of ERβ ablation that set in much later, at 5 months of age [41].

Animals were maintained on a 14 h light, 10 h dark light cycle in standard polycarbonate caging with Sani-chip bedding (Irradiated Aspen Sani-chip; P.J. Murphy Forest Products Corp. Montville, NJ) to eliminate possible corn-based mycoestrogen exposure. All animals are fed *ad libitum* Teklad diet 2020 (Harlan Laboratories Inc.) which lacks soybean meal, alfalfa or animal products that might complicate the study by introduction of uncontrolled levels of estrogenic compounds. Sterile drinking water was generated by a dedicated water purification system (Millipore Rios 16 with ELIX UV/Progard 2) that reduces oxidizable organics to less than 1% of source levels. Drinking water was dispensed from glass water bottles. Concentrations of BPA in drinking water and all experimental reagents were confirmed below the level of detection of a BPA-specific and extremely sensitive ELISA based assay with a minimum quantitative detection limit of 0.05 ng/ml [1].

### Isolation and culturing of ventricular myocytes

Ventricular myocytes from rodent hearts were enzymatically dissociated using Langendorff perfusion with a Tyrode solution composed of (mM) NaCl 118, KCl 5.4, HEPES 10, NaH_2_PO_4_ 0.33, MgCl_2_ 2, glucose 10 (pH = 7.4) and containing 0.7 mg/ml or 1 mg/ml type II collagenase (Worthington Biochem, Lakewood, NJ) for rat and mouse hearts, respectively. For rat myocyte dissociation, the solution also contained 1 mg/ml BSA, 0.2 mg/ml hyaluronidase, and 0.025 mM CaCl_2_. Isolated ventricular myocytes were suspended in 1.0 mM Ca^2+^-Tyrode solution and allowed to sediment by gravity. Supernatant was then removed and myocytes were resuspended. This process was repeated 2–3 times to enrich rod-shaped live myocytes and remove dead cells and non-myocytes. Isolated rat myocytes were suspended in a Medium-199 based solution (Gibco, catalog no. 31100-035, with Earle's salts and L-glutamine, without NaHCO_3_, lot no. 561846; prepared using BPA-free water), plated on laminin (Sigma-Aldrich, CAS 114956-81-9, various lots)-coated glass coverslips at a density of approximately 5×10^4^ cells/cm^2^ in 35×10 mm polystyrene culture dishes, and allowed to adhere for 10 minutes at 37°C. Dead myocytes did not adhere to the cover slips and were rinsed off with additional medium. Myocytes were cultured in 2 ml medium/dish in 5% CO_2_ at 37°C in a humidified incubator for 24 hours prior to analysis. Resulting cultures typically contained >95% myocytes.

### Myocyte calcium transient and spark analysis

Isolated ventricular myocytes were loaded with fluo-4 acetoxymethyl ester (5 µM; Molecular Probes, Eugene, OR) for 15 min at room temperature, and then washed with Tyrode's solution. Myocytes were placed in Tyrode solution (with 1.8 mM CaCl_2_) containing either vehicle or treatment drug, in a custom made glass-plexiglass recording chamber at room temperature (24°C). Analysis of Ca^2+^ transients and sparks was made between 2 to 7 minutes following treatment. Ca^2+^ sparks were recorded from quiescent, unstimulated myocytes. For Ca^2+^ after-transient recording, myocytes were paced with field stimulation (Grass S48 stimulator, Grass Instruments, Quincy, MA) for 8 seconds at a rate of 2 Hz with 2 ms 1.5x threshold pulses. The presence of any spontaneous Ca^2+^ after-transients following pacing was recorded. Fluorescence images were recorded using a Zeiss LSM 510 inverted confocal microscope through a 40x water-immersion objective lens with excitation wavelength of 488 nm. Fluorescence signals were measured at >515 nm with line-scan imaging at 3.07 ms intervals, with each line comprising 512 pixels spaced at 0.056 µm. Image processing and data analysis were performed using IDL software (ITT Visual Information Solutions, Boulder, CO). For analysis of the amplitude and kinetics of field-stimulated or caffeine-induced Ca^2+^ transients, fluorescence signals were measured from fluo-4 loaded myocytes using a Nikon TE 2000 microscope and an InCyt Standard PM photometry system (Intracellular Imaging, Cincinnati, OH). Field stimulated Ca^2+^ transients were elicited with 2 ms 1.5x threshold pulses at a rate of 0.5 Hz. For recording of caffeine-induced Ca^2+^ transients, myocytes were field stimulated at 0.5 Hz for 20 seconds, and were exposed to solution containing 10 mM caffeine immediately following cessation of pacing.

### Surface electrocardiography (ECG) from rat hearts

Following dissection, hearts were quickly cannulated via the aorta, and retrograde perfused on a Langendorff apparatus with 37°C Krebs-Henseleit solution containing (mM) NaCl 118, KCl 4.7, MgSO_4_ 1.2, KH_2_PO_4_ 1.2, EDTA 0.5, CaCl_2_ 2.5, NaHCO_3_ 25, and glucose 11, pH = 7.4, bubbled with 95% O_2_ and 5% CO_2_, at a pressure of 80 mmHg and perfusion rate of ∼15 ml/min. Hearts were perfused under control conditions for at least 1 hour to allow stabilization before exposure to solution containing treatment drug(s). Electrocardiograph was continuously measured from the surface of the heart, with electrodes positioned at the base and apex of the heart. Data collection and analysis were performed using the Powlab 4/30 data acquisition system and LabChart 7 software (AD Instruments, Colorado Springs, CO).

### Myocyte after-contraction analysis

Myocytes were placed in a plexiglass cell chamber filled with 1.8 mM CaCl_2_-Tyrode solution at room temperature (24°C). The solution also contained either vehicle or various treatment drugs. Myocyte contraction was recorded between 2 to 7 minutes following treatment. Myocytes were excited with field stimulation (Grass S48 stimulator, Grass Instruments, Quincy, MA) with 2 ms 1.5x threshold pulses at a rate of 0.5 Hz. Steady state myocyte shortening was imaged with a CCD camera and examined using a video-edge detector (Crescent Electronics, Sandy, UT). Data were sampled through an Axon Digidata 1322A board using the PCLAMP 9 software (both from Molecular Devices, Sunnyvale, CA).

### Patch clamp recording of the L-type Ca^2+^ current

The L-type Ca^2+^ current was recorded at room temperature (24°C) using the whole-cell patch clamp technique with an Axonpatch-200B amplifier (Axon Instruments, Foster City, CA). Extracellular solution contained (in mM) TEA-Cl 137, CsCl 5.4, CaCl_2_ 2, MgCl_2_ 1, HEPES 5, glucose 10, and 4-aminopyridine 3 (pH = 7.4). Glass pipettes were filled with solution containing (in mM) Cs-aspartate 115, CsCl 20, EGTA 11, HEPES 10, MgCl_2_ 2.5, Mg-ATP 2 and Na-GTP 0.1 (pH = 7.2), and had a resistance of 1.5–2.5 MΩ. After the membrane was ruptured, cells were clamped at –50 mV for 5 minutes to allow dialysis of the intracellular solution and stabilization of the Ca^2+^ currents before measurement began. Data collection and analysis were performed using PCLAMP 9 software.

### Statistical analysis

All experiments were independently repeated using myocytes isolated from at least 4 hearts. Statistical analysis was conducted using a paired or unpaired t-test, or one-way or 2- way (e.g. sex vs. treatment) analysis of variance (ANOVA) with differences between treatment groups assessed using a multiple comparison post-test. Frequency of events (e.g., percentage of myocyte with triggered activities) was analyzed using a chi-square (χ^2^) test. Minimal level of statistical significance for differences in values is considered to be p<0.05 (*). Data was analyzed with SigmaPlot 11.0, Excel and GraphPad Prism® version 5.0.
